# Hormonal responses to a stress load and state anxiety, mood, tiredness, and recovery in Portuguese police cadets

**DOI:** 10.47626/1679-4435-2023-1161

**Published:** 2025-01-31

**Authors:** Maria Rosário Abrantes, Raquel Barreto Madeira, Luís Fernandes Monteiro, Catarina N. Matias, Luís Miguel Massuça

**Affiliations:** 1 Faculty of Physical Education and Sports, Universidade Lusófona, Lisbon, Portugal; 2 Research Center in Sport, Physical Education, Exercise, and Health, Universidade Lusófona, Lisbon, Portugal; 3 ICPOL Research Center, Instituto Superior de Ciências Policiais e Segurança Interna, Lisbon, Portugal

**Keywords:** testosterone, law enforcement officer, stress, physiological, stress, psychological, testosterona, polícia, estresse fisiológico, estresse psicológico

## Abstract

**Introduction:**

Police officers are repeatedly exposed to conditions and incidents unique to their work.

**Objectives:**

To describe the hormonal response to a stress load (circuit fitness test) in police cadets, and to verify the association of hormone responses (cortisol and testosterone) with state anxiety, mood, tiredness, and recovery.

**Methods:**

This is an analytical, observational, cross-sectional study with a quantitative approach. A total of 31 police cadets (all male; age 21.0 ± 4.4 years) were evaluated on February 4, 2022, at Instituto Superior de Ciências Policiais e Segurança Interna, Lisbon, Portugal, in four dimensions: morphological, fitness for police work (simulated circuit), hormonal (cortisol and testosterone responses), and psychological (anxiety, mood, stress, and recovery).

**Results:**

We observed differences in cortisol concentration upon awakening and the reference value; a decrease in cortisol and testosterone concentrations until the time of fitness testing; and increases in cortisol and testosterone concentrations in response to completing the fitness test. Regarding the hormone response in anticipation of the fitness test, there is a direct correlation of cortisol with hostility and social stress and an inverse correlation of testosterone with conflicts/pressure, sleep quality, emotional exhaustion, and the sum of the recovery and stress scales. Regarding the hormone response to actually performing the task, a positive correlation was found between cortisol and personal acceptance and self-regulation.

**Conclusions:**

These findings suggest that the police cadets exhibited a healthy response to the proposed stress situation. However, whether they would respond likewise in real-world scenarios is unclear.

## Abstract (pt)

**Introdução:**

Policiais estão repetidamente expostos a condições e incidentes exclusivos do seu trabalho.

**Objetivos:**

Descrever a resposta hormonal a uma carga de estresse (circuito) em cadetes-alunos de polícia portugueses e verificar a associação das respostas hormonais (cortisol e testosterona) com os estados de ansiedade, humor, cansaço e recuperação.

**Métodos:**

Trata-se de um estudo analítico, observacional, transversal e de abordagem quantitativa, em que participaram 31 cadetes-alunos de polícia do sexo masculino (idade, 21,0±4,4 anos). Eles foram avaliados (Instituto Superior de Ciências Policiais e Segurança Interna, 4 de fevereiro de 2022) nas dimensões morfológica, de aptidão para a função, hormonal (cortisol e testosterona) e psicológica (estados de ansiedade e de humor, estresse e recuperação).

**Resultados:**

Observaram-se diferenças entre a concentração de cortisol ao acordar e o valor de referência; diminuições nas concentrações de cortisol e testosterona até o momento de realização do circuito; e aumentos das concentrações de cortisol e testosterona em resposta à realização do circuito de aptidão para a função policial. Relativamente à resposta hormonal de antecipação ao circuito, foi observada uma correlação direta do cortisol com a hostilidade e com o estresse social e uma correlação inversa da testosterona com conflitos/pressão, qualidade do sono, exaustão emocional e a soma das escalas de recuperação e estresse. No que diz respeito à resposta hormonal à realização da tarefa, observou-se uma correlação positiva do cortisol com a aceitação pessoal e a autorregulação.

**Conclusões:**

Os resultados sugerem que os cadetes-alunos de polícia responderam de forma saudável à situação de estresse proposta. Contudo, não fica claro se as respostas seriam as mesmas em cenários reais.

## INTRODUCTION

Police officers are repeatedly exposed to conditions and incidents unique to their work, which can be perceived and responded to as stressful.^1^ This population often experiences a state of psychological inflexibility due to the multiplicity of difficult, challenging demands faced in police work.^2^ However, the ability to regulate emotional action depends, largely, on control exerted by the prefrontal cortex over the amygdala,^3^ although it is unclear whether this relationship holds for police officers, who may have strong compensatory frontal control over their emotional tendencies.

The stress response depends on a set of brain regions and peripheral systems, which communicate in a complex, coordinated interaction, providing a conscious understanding of one’s environment and the ability to interact with it.^4^

After sensory identification of stimuli, emotional and cognitive assessments can update response selection, taking into account experience and setting.^5^

The stress response is an interlinked, two-stage system involving the hypothalamic-pituitary-adrenal (HPA) axis and the autonomic nervous system (ANS). Psychological studies in humans have recorded changes in cortisol and testosterone levels in response to challenges or stressful scenarios,^6^ highlighting that these two hormone systems can be mutually modulating and that their interactions have consequences for subsequent behaviors or psychological traits.

To address the complex reality of modern-day police operations, the training of police cadets includes exercise circuits based on critical operational tasks^7^ which can drastically alter the physiological and psychological condition of police officers,^8^ e.g., by triggering release of stress hormones.^9^

Within this context, the present study was designed to: (i) describe the hormonal response to a stress load (performance on a physical fitness test circuit designed to assess readiness to perform police duties) in Portuguese police cadets; and (ii) verify the association of hormone responses (cortisol and testosterone) with state anxiety, mood, tiredness, and recovery.

## METHODS

### STUDY DESIGN

This analytical, observational, and cross-sectional study was approved by the Ethics Committee of the Faculty of Physical Education and Sport of Universidade Lusófona, Lisbon, Portugal (opinion no. 224/2022) and conducted in accordance with the standards for human subject research set forth in Declaration of Helsinki.^10^ All male cadets of the XXXVII and XXXVIII Police Officer Training Courses (*Cursos de Formação de Oficiais de Polícia*, CFOP) of the Higher Institute of Police Sciences and Internal Security (ISCPSI) in Lisbon, Portugal, were invited to take part in the study. Before any study procedures, the objectives, design, data collection methods, and all potential risks and benefits of the study were explained to the volunteers. All cadets who agreed to participate provided verbal and written informed consent prior to enrollment.

### SAMPLE

The study sample comprised 31 male cadets of the XXXVIII CFOP (first-year) and XXXVII CFOP (second-year) courses taught at ISCPSI (representing approximately 51% of all cadets), aged 18 to 32 years (mean ± SD, 21.00±4.37 years). The profile of the participants is described in the Results section.

### ASSESSMENT PROCEDURES AND INSTRUMENTS

**Figure 1:**
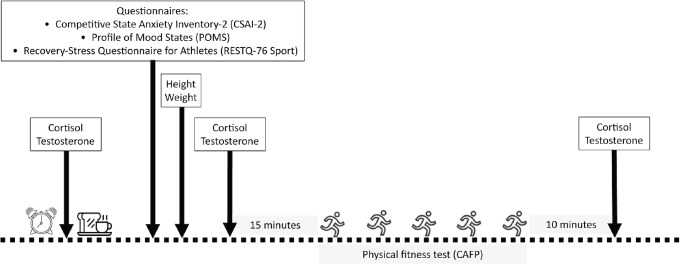
Study design.

Participants underwent four assessments: (i) morphological evaluation (height, body mass, and body mass index [BMI]); (ii) assessment of fitness for police duty; (iii) hormonal assessment; and (iv) psychological assessment (state anxiety, mood, stress, and recovery). The study design is presented in Figure 1, as well as a more detailed description of the assessments conducted for each of the dimensions of interest.

### MORPHOLOGICAL ASSESSMENT

Body mass (Seca model 761 7019009 scales, resolution 0.5 kg) and height (Siber-Hegner anthropometric kit, resolution 0.1 cm; technical error of measurement, R ≥ 0.98) were measured in accordance with the International Society for the Advancement of Kinanthropometry guidelines.^11^ The BMI was then calculated as follows: BMI = (kg/m^2^).

### ASSESSMENT OF FITNESS FOR POLICE DUTIES

**Figure 2:**
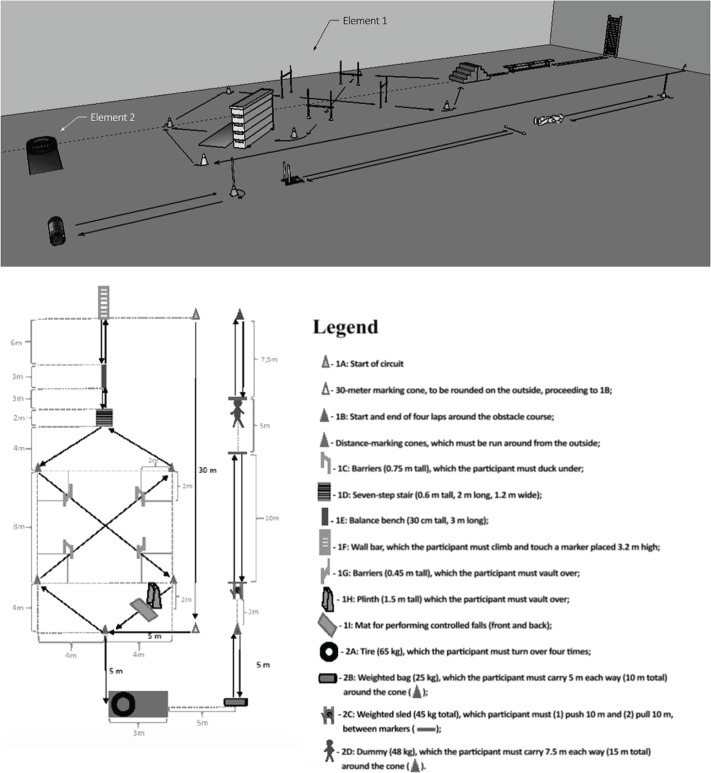
Layout (3D and 2D) and components of the CAFP.^12^

The Police Duty Fitness Circuit (*circuito de aptidão para a função policial,* CAFP) is a physical fitness test composed of tasks that aim to simulate: (i) a foot pursuit/deployment to the scene of a call-out; and (ii) resolution of the scenario. The layout of the CAFP and a description of its tasks are given in Figure 2. A more detailed description of the CAFP is available in Teixeira et al.^12^

### HORMONE ASSESSMENT

For assessment of hormonal response, cortisol and testosterone concentrations were measured in saliva samples collected into Salivette tubes, designed specifically for this purpose (Sarstedt, Germany), at the following time points: (i) upon waking (T0); (ii) immediately before the CAFP (T1, approximately 15 minutes before); and (iii) 10 minutes after completion of the CAFP (T2). All salivary specimen collection procedures were conducted to as to minimize variance between samples and/or methodological errors. Participants were instructed to refrain from eating or drinking for at least 2 hours before sample collection, as foods or beverages rich in sugar, caffeine, alcohol, or acidity may stimulate salivary flow or alter pH levels in the oral cavity, jeopardizing antibody antigen binding and enzyme activity and thus invalidating the results of assays subsequently performed on the specimens. They were, however, encouraged to drink water as needed to maintain adequate hydration.

At the time of collection, participants were asked to place the Salivette cotton swab within the oral cavity, against the cheek mucosa, for at least 2 minutes, until it was thoroughly saturated with saliva. The cotton swab was placed back into the Salivette tube, the cap was placed onto the tube and the date, time, and participant ID were recorded. After collection, saliva specimens were frozen at a minimum temperature of -20 °C for subsequent biochemical assays. Specimens were thawed overnight before the day of analysis and were simultaneously tested by enzyme-linked immunosorbent assay (ELISA). The Expanded Range High Sensitivity Salivary Cortisol Enzyme Immunoassay Kit and Expanded Range Salivary Testosterone Enzyme Immunoassay Kit (Salimetrics, USA) were used.

Finally, hormone concentrations were recorded (i) upon waking (T0), (ii) before completing the CAFP (T1), and (iii) after completing the CAFP (T2). Furthermore, hormone responses to (i) pre-CAFP activation (∆T1-T0) and (ii) the stress induced by CAFP performance (∆T2-T1) were calculated.

### PSYCHOLOGICAL ASSESSMENT

Psychological assessment was carried out before the CAFP and included evaluation of: (i) competitive state anxiety; (ii) mood profile; and (iii) frequency of current stress symptoms, as well as frequency of activities associated with recovery.

The *Inventário do Estado de Ansiedade Competitiva* (IEAC) is a version of the Competitive State Anxiety Inventory-2 (CSAI-2), translated into Portuguese and adapted by Cruz & Viana.^13^ Respondents score each of the 27 items on a scale from 1 (“not at all”) to 4 points (“very much”). Results for each subscale are obtained by the sum of the values assigned to each of the respective items; higher scores reflect higher levels of: (i) cognitive anxiety; (ii) somatic anxiety; and (iii) self-confidence.

The *Perfil de Estados de Humor* questionnaire is a Portuguese adaptation^14^ of the Profile of Mood States (POMS) questionnaire, which a self-reported psychometric measure of mood states consisting of a 65-item checklist of adjectives related to mood. Each adjective on the list is scored from 0 (none) to 4 (very), based on how well each item describes the participant’s mood during the past week (including the day of assessment itself). Following an established standard scoring method, six mood scales are calculated: (i) tension-anxiety (T); (ii) depression-dejection (D); (iii) anger-hostility (H); (iv) vigor-activity (V); (v) fatigue-inertia (F); and (vi) confusion-bewilderment (C). A higher score denotes a higher level of the corresponding emotion. In addition, two other variables were calculated: (i) the sum of the scales; and (ii) the total mood disturbance (TMD) score, given by the formula ([T + D + H + F + C] - V) + 100.

The Recovery Stress Questionnaire for Athletes (RESTQ-76 Sport), translated and validated for Portuguese by Costa & Samulski,^15^ is designed to measure the frequency of current stress symptoms, as well as the frequency of activities associated with recovery. This questionnaire covers a specific period of 3 or 7 days/nights, and is scored on a Likert-type scale from 0 (never) to 6 (always), indicating how often the participant performed the corresponding activities or experienced the corresponding states.

The instrument consists of 77 items (one of which, item 1, is a “warm-up” item and is thus not included in the result), organized into 19 scales: (i) general stress; (ii) emotional stress; (iii) social stress; (iv) conflicts/pressure; (v) fatigue; (vi) lack of energy; (vii) physical complaints; (viii) success; (ix) social recovery; (x) physical recovery; (xi) general well-being; (xii) sleep quality; (xiii) disturbed breaks; (xiv) emotional exhaustion; (xv) injury; (xvi) being in shape; (xvii) personal accomplishment; (xviii) self-efficacy; and (xix) self-regulation. The sum of the scales was also calculated as a final variable.

### STATISTICAL ANALYSIS

Descriptive statistics - namely, measures of central tendency (mean) and dispersion (standard deviation, SD) - were used to characterize the sample.

Regarding statistical inference procedures, the one-sample Student’s *t* test was used to ascertain whether baseline cortisol and testosterone levels were within reference values (cortisol, 0.5 µg/dL; testosterone, 156.5 pg/mL), while Student’s *t* test for paired samples was used to assess the magnitude of hormonal responses (as it was not necessary to ensure homogeneity of variances). The assumption of normality was validated by the Kolmogorov-Smirnov test (KS[31]_cortisol-T0_ = 0.157, p = 0.051; KS[31]_cortisol-T1_ = 0.098, p = 0.200; KS[31]_cortisol-T2_ = 0.156, p = 0.053; KS[31]_testosterone-T0_ = 0.134, p = 0.167; KS[31]_testosterone-T1_ = 0.217, p < 0.001; KS[31]_testosterone-T2_ = 0.212, p = 0.001).

In addition, measures of association were used to quantify the intensity and direction of the relationship between hormone responses (T1; T2; ∆T1-T0; ∆T2-T1) and psychological attributes (Spearman’s correlation coefficient was used).

Statistical analysis was performed in the Statistical Package for the Social Sciences (SPSS) Version 28.0 software package (Armonk, NY). The significance level was set at p < 0.05 for all tests.

## RESULTS

On assessment of 31 male police cadets (mean age, 21.00±4.37 years; height, 1.77±0.05 m; body mass, 72.14±7.60 kg; BMI, 22.55±2.84 kg/m^2^), we found: (i) differences between the cortisol concentration upon awakening (T0) and the reference value (t[30] = 2.643, p = 0.013, d = 0.475), with no such differences in testosterone concentrations (t[30] = 1.993, p = 0.055, d = 0.358); (ii) decreases in cortisol (-0.35 µg/dL, t[30] = 8.716, p < 0.001) and testosterone (-52.55 pg/mL, t[30] = 10.979, p < 0.001) before performance of the CAFP; and (iii) increases in cortisol (0.10 µg/dL, t[30] = -5.014, p < 0.001) and testosterone (16.31 pg/mL, t[30] = -5.130, p < 0.001) concentrations in response to the CAFP. The results are shown in Table 1.

**Table 1:** Salivary cortisol and testosterone concentrations upon awakening (T0), pre-CAFP (T1), and post-CAFP (T2), as well as differences between collection time points (∆T1-T0; ∆T2-T1)

	Mean	SD	Minimum	Maximum
Cortisol (µg/dL)				
On waking (T0)	0.62^*^	0.25	0.17	0.93
Pre-CAFP (T1)	0.27	0.11	0.11	0.53
Post-CAFP (T2)	0.38	0.16	0.14	0.76
∆T1-T0	-0.35	0.22	-0.73	-0.04
∆T2-T1	0.10	0.12	0.03	0.55
Testosterone (pg/mL)				
On waking (T0)	166.62	28.27	116.00	208.00
Pre-CAFP (T1)	114.06	22.59	88.00	176.00
Post-CAFP (T2)	130.37	25.39	100.00	184.50
∆T1-T0	-52.55	26.65	-103.00	-6.00
∆T2-T1	16.31	17.70	-12.00	64.00

SD = standard deviation.

* p < 0.05 (versus mean reference value: 0.5 µg/dL).

Correlations were found between cognitive anxiety e pre-CAFP cortisol (r = 0.496, p = 0.005), and post-CAFP cortisol (r = 0.504, p = 0.004). Regarding mood states: (i) hostility showed an inverse correlation with cortisol on waking (r = -0.390, p = 0.030), but a direct correlation with the anticipatory response to the CAFP (r = 0.356, p = 0.049); (ii) fatigue and the sum of all scales correlated with pre-CAFP (r = 0.614, p < 0.001; r = 0.430, p = 0.016) and post-CAFP(r = 0.430, p = 0.002; r = 0.373, p = 0.039) cortisol concentrations. Finally, regarding recovery and stress: (i) general stress (r = 0.414, p = 0.021) and being in shape (r = -0.473, p = 0.007) directly correlated with pre-CAFP cortisol concentrations; (ii) social stress (r = 0.353, p = 0.051) showed a marginal correlation with the anticipatory response to the CAFP; and (iii) personal accomplishment (r = 0.365, p = 0.044) and self-regulation (r = 0.357, p = 0.049) correlated with the hormonal response to performing the actual task (CAFP). The results are shown in Table 2.

**Table 2:** Descriptive statistics of the scales that characterize state anxiety, mood, recovery, and stress and correlations (Spearman’s rho) of these scales with cortisol concentrations (T0, T1, T2) and variations between these concentrations at different time points (∆T1-T0; ∆T2-T1) in response to a test of physical fitness for police duties

	Mean	SD	Correlations^*,†^				
			T0 (on waking)	T1 (pre-CAFP)	T2 (post-CAFP)	∆T1-T0	∆T2-T1
State anxiety							
Cognitive anxiety	11.61	4.27	0.013	0.496‡	0.504‡	0.157	0.199
Somatic anxiety	15.52	4.95	-0.192	0.225	0.038	0.313	-0.270
Self-confidence	28.29	7.18	-0.066	-0.345	-0.251	-0.004	0.047
Mood states							
Tension	6.29	3.06	-0.032	0.328	0.137	0.204	-0.146
Depression	1.97	3.68	-0.173	0.092	0.118	0.187	0.049
Hostility	2.03	2.73	-0.390^§^	-0.057	-0.060	0.356^§^	-0.037
Vigor	14.68	4.13	0.109	-0.106	-0.073	-0.130	0.054
Fatigue	6.03	4.92	0.169	0.614^||^	0.545‡	-0.015	0.084
Confusion	6.03	2.17	-0.077	-0.083	0.117	0.070	0.338
Sum of scales	37.03	10.95	-0.026	0.430§	0.373§	0.156	0.081
Total mood disturbance	107.68	13.66	-0.132	0.308	0.256	0.212	-0.019
Recovery and stress							
General stress	5.45	3.87	-0.010	0.414§	0.299	0.177	-0.052
Emotional stress	7.19	3.25	-0.074	0.249	0.144	0.227	-0.108
Social stress	6.13	3.44	-0.197	0.251	0.092	0.353§	-0.241
Conflicts/pressure	9.61	3.78	0.038	0.151	0.200	0.002	0.180
Fatigue	10.97	3.75	0.212	0.314	0.287	-0.133	-0.059
Lack of energy	7.68	3.66	-0.129	0.025	0.000	0.164	-0.073
Physical complaints	7.03	2.94	-0.038	0.200	0.159	0.124	-0.030
Success	15.26	2.82	-0.011	-0.337	-0.094	-0.152	0.201
Social recovery	16.68	3.00	-0.114	-0.019	0.120	0.148	0.239
Physical recovery	14.77	2.69	-0.066	-0.336	-0.202	0.030	0.034
General well-being	16.68	2.68	-0.114	-0.323	-0.042	0.006	0.294
Sleep quality	10.00	2.90	-0.066	0.037	0.051	0.125	0.055
Disturbed breaks	9.58	4.37	0.185	0.318	0.248	-0.110	-0.105
Emotional exhaustion	5.45	3.89	-0.072	0.286	0.105	0.162	-0.234
Injury	9.00	3.71	-0.209	0.271	0.209	0.316	-0.082
Being in shape	13.97	3.72	-0.068	-0.473‡	-0.250	-0.058	0.125
Personal accomplishment	15.19	3.28	0.224	-0.043	0.176	-0.245	0.365§
Self-efficacy	15.19	3.35	-0.111	-0.330	-0.137	0.010	0.176
Self-regulation	15.03	3.75	0.190	-0.177	0.064	-0.271	0.357§
Sum of scales	210.87	30.23	-0.146	0.123	0.081	0.226	0.007

SD = standard deviation.

* The estimate is based on Fisher’s r-to-z transformation.

† The estimate of standard error is based on the formula proposed by Fieller, Hartley, and Pearson.

‡ p < 0.01.

§ p < 0.05.

|| p < 0.001.

Additionally, there were no correlations between testosterone responses and state anxiety or mood. However, regarding recovery and stress, we found that: (i) pre-CAFP testosterone concentration correlated inversely with sleep quality (r = -0.372, p = 0.039) and with the sum of scales (r = -0.372, p = 0.039); and (ii) the anticipatory hormonal response to the CAFP correlated inversely with conflicts/pressure (r = -0.356, p = 0.049), sleep quality (r = -0.405, p = 0.024), emotional exhaustion (r = -0.358, p = 0.048), and the sum of the recovery and stress scales (r = -0.580, p < 0.001). The results are shown in Table 3.

**Table 3:** Descriptive statistics of the scales that characterize state anxiety, mood, recovery, and stress and correlations (Spearman’s rho) of these scales with testosterone concentrations (T0, T1, T2) and variations between these concentrations at different time points (∆T1-T0; ∆T2-T1) in response to a test of physical fitness for police duties

	Mean	SD	Correlations^*,†^				
			T0 (on waking)	T1 (pre-CAFP)	T2 (post-CAFP)	∆T1-T0	∆T2-T1
State anxiety							
Cognitive anxiety	11.61	4.27	-0.061	0.009	0.099	-0.053	0.181
Somatic anxiety	15.52	4.95	0.122	-0.221	0.077	-0.304	0.286
Self-confidence	28.29	7.18	-0.093	-0.035	-0.035	0.017	0.085
Mood states							
Tension	6.29	3.06	0.320	0.055	0.261	-0.322	0.263
Depression	1.97	3.68	0.092	0.103	0.153	-0.025	0.061
Hostility	2.03	2.73	0.256	0.119	0.200	-0.260	0.114
Vigor	14.68	4.13	0.028	-0.192	-0.197	-0.073	0.034
Fatigue	6.03	4.92	-0.008	0.011	0.022	-0.033	-0.038
Confusion	6.03	2.17	0.091	-0.097	0.131	-0.152	0.210
Sum of scales	37.03	10.95	0.158	-0.028	0.075	-0.252	0.133
Total mood disturbance	107.68	13.66	0.106	0.118	0.155	-0.092	0.016
Recovery and stress							
General stress	5.45	3.87	0.003	-0.282	-0.100	-0.235	0.165
Emotional stress	7.19	3.25	0.184	-0.122	0.115	-0.219	0.203
Social stress	6.13	3.44	0.058	-0.308	-0.027	-0.247	0.271
Conflicts/pressure	9.61	3.78	0.147	-0.189	0.004	-0.356‡	0.219
Fatigue	10.97	3.75	0.312	0.171	0.159	-0.064	-0.021
Lack of energy	7.68	3.66	0.223	-0.094	-0.081	-0.219	-0.110
Somatic complaints	7.03	2.94	-0.086	-0.146	-0.191	-0.078	-0.115
Success	15.26	2.82	-0.003	-0.023	-0.096	0.021	-0.064
Social recovery	16.68	3.00	-0.041	-0.096	0.040	-0.132	0.164
Physical recovery	14.77	2.69	0.051	-0.186	-0.015	-0.190	0.280
General well-being	16.68	2.68	-0.106	-0.079	-0.109	-0.035	0.033
Sleep quality	10.00	2.90	0.090	-0.372‡	-0.192	-0.405‡	0.145
Disturbed breaks	9.58	4.37	0.226	-0.005	0.104	-0.169	-0.006
Emotional exhaustion	5.45	3.89	0.075	-0.323	-0.078	-0.358‡	0.236
Injury	9.00	3.71	-0.055	-0.257	-0.158	-0.143	0.035
Being in shape	13.97	3.72	-0.023	-0.290	-0.161	-0.214	0.222
Personal accomplishment	15.19	3.28	0.064	0.011	-0.029	-0.087	-0.050
Self-efficacy	15.19	3.35	0.050	-0.178	-0.098	-0.219	0.230
Self-regulation	15.03	3.75	0.214	-0.018	0.026	-0.227	0.141
Sum of scales	210.87	30.23	0.244	-0.372‡	-0.114	-0.580§	0.271

SD = standard deviation.

^*^ The estimate is based on Fisher’s r-to-z transformation.

† The estimate of standard error is based on the formula proposed by Fieller, Hartley, and Pearson.

‡ p < 0.05.

§ p < 0.001.

## DISCUSSION

In this study, the hormones cortisol and testosterone exhibited a typical pattern of secretion upon awakening (Table 1) and in response to a stress load (CAFP). Although mean cortisol values on waking did not correspond to the mean reference value for the technique employed (0.5 µg/dL), they can be considered to fall within healthy/non-pathological range (0.112-0.743 µg/dL).

### HORMONE RESPONSES

Although cortisol levels increase during sleep, most individuals experience a marked increase in secretion of this hormone (beyond the circadian pattern) immediately on waking; this is known as the cortisol awakening response (CAR), and precedes a gradual, sustained decline in hormone levels throughout the day.^16^

The CAR values recorded by Tavares et al.^17^ (0.578 µg/dL) - with the authors associating these values to the affinity of military personnel with their duties - are consistent with those found in the present study.

The functional role of the CAR is still the object of debate, but it has been hypothesized to assist with spontaneous memory retrieval as neural networks shift from sleep to the waking state,^16^ as well as with emotional preparation for the day ahead.^18^ The magnitude of the CAR was found to be associated with acute and chronic stress.^18^ Conversely, the literature also reports that a blunted CAR is associated with peritraumatic distress or dissociation.^19^

The mean testosterone values upon waking measured in this study were within the reference range for the technique employed (156.5 pg/mL). Although testosterone also exhibits peak levels on awakening (usually in the morning) and trough levels before bed (usually at night), it is less clear whether abrupt changes in testosterone levels occur upon waking, in a manner analogous to the RCA. Although this pattern of morning elevation and evening reduction in testosterone levels has been well described, the functional significance of its diurnal variation is comparatively poorly understood. However, given the evidence of coregulation between cortisol and testosterone, one possibility is that testosterone rises transiently upon waking to support the same functions as cortisol does. Alternatively, a change in metabolic priorities may facilitate a decline in testosterone levels as cortisol rises.

Unlike in research into the role of the HPA axis and cortisol, few studies have employed protocols capable of characterizing the change in testosterone levels which occurs upon awakening.^20^ Despite evidence of coregulation and an interrelationship between the HPA and hypothalamic-pituitary-gonadal (HPG) axes,^20^ whether changes in testosterone levels on waking may correlate with (or be modulated by) simultaneous changes in cortisol secretion (i.e., the CAR) remains unclear. However, it is established that: (i) acute cortisol production in response to severe stress can suppress the function of testosterone^21^; and (ii) cortisol and testosterone concentrations tend to correlate positively throughout the day, pointing to likely roles of these hormones in the coordination of metabolism and behavior.^20^ Hence, the findings of the present study are consistent with the literature insofar as they showed a decline in cortisol and testosterone values between the time of saliva collection on waking and the next time point of collection (pre-CAFP).

Regarding hormonal responses to the stress induced by the CAFP, concentrations of both cortisol and testosterone were found to increase (Table 1). These results are in accordance with a recent study by Dergaa et al.,^22^ which found that short-term maximal exercise significantly influenced cortisol and testosterone levels in a sample of young police officers: (i) anaerobic exercise increased cortisol levels by 35% (12% of this increase occurred during the recovery period); and (ii) aerobic exercise increased cortisol levels by 54%. Thus, the increase in cortisol concentration after the physical fitness test circuit can be explained by the role of this hormone in regulating metabolism during exercise.^23^

Regarding testosterone concentration, Dergaa et al.^22^ did not observe significant changes after short-term maximal exercise. However, Akinola & Mendes^23^ had reported increases in both cortisol and testosterone concentrations after a simulation with police officers, corroborating the results observed in the present study with Portuguese police cadets.

### HORMONE RESPONSES, ANXIETY, MOOD, FATIGUE, AND RECOVERY

Secondarily, we sought to ascertain whether associations existed between changes in cortisol levels and changes in testosterone levels during the CAFP.

Lieberman et al.^24^ confirmed activation of the HPA axis by observing, in a training scenario involving 60 U.S. military personnel from the Survival, Evasion, Resistance, and Escape (SERE) school, increases in cortisol levels and suppression of testosterone release. These findings highlight that such changes are recognized as part of the cascade of physiological and psychological events associated with stress, which restrict energy availability.

In the present study, testosterone levels increased from the beginning to the end of the fitness test circuit, demonstrating a positive response to the challenge imposed and a desire to perform well, which rejects the previously described mental stress hypothesis. However, the characterization of police cadets’ psychological response to stress allowed us to highlight (i) the association between cognitive anxiety and preand post-CAFP cortisol levels (meaning that the perception of anxiety, as measured by a questionnaire, is noticeable when cortisol values are increased); and (ii) that no association was observed between somatic anxiety and self-confidence. This highlights that cognitive anxiety and somatic anxiety represent opposite poles in a continuum of cognitive assessment, with self-confidence seen as the absence of cognitive anxiety (or, conversely, cognitive anxiety seen as a lack of self-confidence).^25^

Martens et al.^25^ applied this distinction to anxiety reactions in sports, noting that (i) cognitive anxiety usually manifests as negative expectations regarding performance and a negative self-assessment, whereas (ii) somatic anxiety manifests as physiological responses including elevated heart rate, dyspnea, muscle tension, clammy hands, and a feeling of “butterflies in one’s stomach”. Although the present study had police cadets rather than athletes as participants, the competitive nature inherent to the CAFP means the findings of Filaire et al. are relevant to our assessment. These authors, in (i) a study of paragliding athletes (n = 10), found that, shortly before the jump, a correlation existed between cognitive anxiety and cortisol responses, with cognitive anxiety having been classified as a performance facilitator^26^; and (ii) in tennis players (n = 16), an early rise in cortisol occurred before a competition, with winners showing lower cognitive anxiety and higher self-confidence values and losers showing higher cortisol levels and somatic anxiety.^27^

Regarding mood states, we found no relationship between total mood disturbance and cortisol or testosterone levels (preor post-CAFP). However, it is noteworthy that cortisol correlates with fatigue (cadets with higher perceived fatigue had higher cortisol levels).

Lieberman et al.^24^ studied (realistic and controlled) simulated captivity of military personnel and found that cognition, mood (tension, depression, hostility, vigor, fatigue, confusion, and total mood disturbance), stress hormones (increased cortisol and decreased testosterone), nutritional status, and heart rate were all simultaneously altered.

Regarding perceived stress, the objective was to evaluate the level of tiredness and stress to which participating cadets would be subject, since the literature shows that, when one is exposed to a training load, higher levels of stress and/or lower levels of recovery are expected; to wit: (i) Coutts et al.^28^ observed, in experienced male triathletes, stress and lower recovery with increasing training loads (which improved after load reduction); (ii) Noce et al.^29^ observed, in female volleyball athletes, low stress and high recovery scores during a competition; and (iii) González-Boto et al.^30^ reported similar findings in swimmers. In addition, in the present study we found that: (i) pre-CAFP cortisol levels correlated directly with general stress and inversely with being in shape; and (ii) pre-CAFP testosterone levels correlated inversely with sleep quality.

It bears stressing that the police cadets who took part in the study were still at a skills assessment stage of their training, pursuing academic approval to ensure a good professional placement and to perform well among their peers and superiors. A competitive nature is also part of social and human development and, therefore, needs to be taken into account during the present study. During their training program, police cadets are subjected to a wide range of stressors (physiological and psychological), and, indeed, correlations were observed between hormonal and psychological variables: (i) pre-CAFP and post-CAFP cortisol levels correlated directly with cognitive anxiety and fatigue; and (ii) pre-CAFP cortisol levels correlated directly with general stress and inversely with being in shape. Regarding testosterone, pre-CAFP levels correlated inversely with sleep quality.

Although this study is relevant and contributes knowledge to a field in which there is still a dearth of research, some limitations must be mentioned. One of the main limitations of studies of this design concerns the collection of specimens for hormone measurement, which, even when done in saliva, are invasive and subject to change by the mere act of collection itself, due to their heightened sensitivity. Furthermore, it is important to note that our understanding of the role of steroid hormones in humans is still incomplete, since their signaling cascades, although widely recognized, have not been fully studied.

## CONCLUSIONS

When faced with stressful situations in a controlled environment, Portuguese police cadets appeared to mount healthy physiological (hormonal) and psychological (perceptual) responses. This suggests they are not at risk of acute or chronic stress syndromes. However, this study could not ascertain whether changes in the hormonal pattern of response to stressful stimuli would occur, nor what these changes would be, in the real-world intervention scenarios these cadets will face as police officers. Therefore, further studies should be conducted to investigate the impact of context and setting on hormonal stress response patterns. The aftermath of police intervention and the possible impacts of these stress responses on the daily lives of cadets and officers must also be taken into account, as such changes in hormone levels may have personal and social repercussions for those involved. Further research should also seek to elucidate whether the tools and techniques used herein (saliva specimen collection and administration of psychometric questionnaires) can promote resilience and well-being in individuals exposed to chronic stress or traumatic events during the performance of police duties.
